# Empagliflozin protects against heart failure with preserved ejection fraction partly by inhibiting the senescence-associated STAT1–STING axis

**DOI:** 10.1186/s12933-024-02366-0

**Published:** 2024-07-23

**Authors:** Ying Shi, Lili Zhao, Jing Wang, Xiankun Liu, Yiming Bai, Hongliang Cong, Ximing Li

**Affiliations:** 1 Tianjin Chest Hospital, Tianjin Medical University, Tianjin, 300070 China; 2 Tianjin Key Laboratory of Cardiovascular Emergency and Critical Care, Tianjin Municipal Science and Technology Bureau, Tianjin, 300222 China; 3 Chest Hospital, Tianjin University, Tianjin, 300072 China; 4 Tianjin Institute of Cardiovascular Disease, Tianjin Chest Hospital, Tianjin, 300222 China; 5 Department of Cardiology, Tianjin Chest Hospital, Tianjin, 300222 China; 6 Department of Cardiac Surgery, Tianjin Chest Hospital, Tianjin, 300222 China

**Keywords:** Empagliflozin, Heart failure, Senescence, STAT1, STING

## Abstract

Heart failure with preserved ejection fraction (HFpEF) is a mortal clinical syndrome without effective therapies. Empagliflozin (EMPA) improves cardiovascular outcomes in HFpEF patients, but the underlying mechanism remains elusive. Here, mice were fed a high-fat diet (HFD) supplemented with L-NAME for 12 weeks and subsequently intraperitoneally injected with EMPA for another 4 weeks. A 4D-DIA proteomic assay was performed to detect protein changes in the failing hearts. We identified 310 differentially expressed proteins (DEPs) (ctrl vs. HFpEF group) and 173 DEPs (HFpEF vs. EMPA group). The regulation of immune system processes was enriched in all groups and the interferon response genes (STAT1, Ifit1, Ifi35 and Ifi47) were upregulated in HFpEF mice but downregulated after EMPA administration. In addition, EMPA treatment suppressed the increase in the levels of aging markers (p16 and p21) in HFpEF hearts. Further bioinformatics analysis verified STAT1 as the hub transcription factor during pathological changes in HFpEF mice. We next treated H9C2 cells with IFN-γ, a primary agonist of STAT1 phosphorylation, to investigate whether EMPA plays a beneficial role by blocking STAT1 activation. Our results showed that IFN-γ treatment caused cardiomyocyte senescence and STAT1 activation, which were inhibited by EMPA administration. Notably, STAT1 inhibition significantly reduced cellular senescence possibly by regulating STING expression. Our findings revealed that EMPA mitigates cardiac inflammation and aging in HFpEF mice by inhibiting STAT1 activation. The STAT1–STING axis may act as a pivotal mechanism in the pathogenesis of HFpEF, especially under inflammatory and aging conditions.

**Graphical abstract:**

The schematic figure depicts a mechanism model of the STAT1–STING axis in HFpEF (this figure was drawn using FigDraw software).

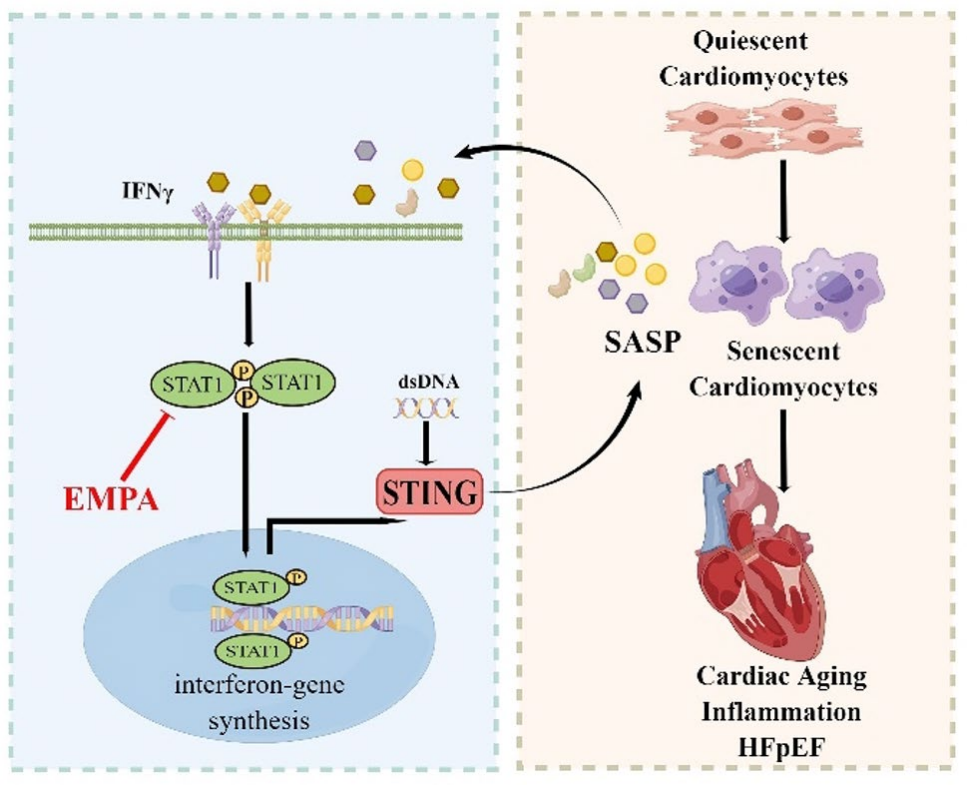

**Supplementary Information:**

The online version contains supplementary material available at 10.1186/s12933-024-02366-0.

## Introduction

Heart failure (HF), a major cause of high morbidity and mortality worldwide, has become a global health problem affecting 64.3 million people [1]. Notably, approximately 50% of patients hospitalized for HF suffer from heart failure with preserved ejection fraction (HFpEF). HFpEF is considered to be a major challenge in cardiology today because of the lack of effective therapies [2]. The incidence of HFpEF may increase to 8% in people aged 65 years, which even exceeds 10% in older people (≥ 75 years old) [3]. Therefore, it is extremely urgent to find innovative treatments to address this ongoing epidemic.

Sodium–glucose cotransporter 2 inhibitors (SGLT2is), a novel class of antidiabetic drugs, have shown favorable cardiovascular outcomes. Encouraging results from clinical trials, such as the EMPEROR‒Preserved and DELIVER trials, have demonstrated that both empagliflozin (EMPA) and dapagliflozin (DMPA) can significantly reduce the risk of cardiovascular death or hospitalization for HF in HFpEF patients [4, 5]. According to clinical trials of SGLT2is showing benefits across the ejection fraction spectrum, SGLT2 inhibitors are now the first therapeutic class to be foundational treatment for HF, regardless of LVEF [6]. Several mechanisms have been proposed to explain their beneficial cardiovascular effects, including myocardial energy metabolism, inflammation, autophagy, and cardiac remodeling [7]. However, the underlying mechanisms by which EMPA protects against HFpEF remain unclear.

Studies have shown that aging can lead to heart deterioration, such as cardiac hypertrophy and diastolic dysfunction. Additionally, aged hearts are predisposed to HFpEF, which often involves increased extracellular matrix deposition and interstitial fibrosis diffusion [8]. Cell senescence refers to irreversible growth arrest accompanied by the upregulated expression of p53, p16^INK4a^, and p21^CIP1/WAF1^ and increased activity of aging-related galactosidase (SA-β-gal). In addition, senescent cells secrete a large number of growth factors, interleukins, chemokines, and matrix metalloproteinases, which are called the senescence-associated secretory phenotype (SASP) [9]. Cellular senescence contributes to HFpEF development by increasing systemic and vascular inflammation [10]. On the other hand, chronic low-grade inflammation is a key feature of aging and age-related diseases [11]. The interplay between inflammation and the aging process drives pathological changes of HFpEF.

Recently, increasing evidence has suggested that SGLT2is can also be repurposed as anti-aging drugs that prevent the extrinsic harmful effects of senescent cells [12]. Several studies have demonstrated that SGLT2is improve overall life expectancy and slow aging by inhibiting multiple pathways, such as oxidative stress, inflammation, mitochondrial dysfunction and the profibrotic pathway [13, 14]. Although the anti-senescence effect of EMPA has been observed in kidney tissue [15], few studies have assessed its role in cardiac aging. In this study, we performed 4D-DIA proteomic analysis to explore the underlying mechanisms by which EMPA improves cardiac function in HFpEF mice and to investigate the effects of EMPA on cardiomyocyte (CM) senescence.

## Methods

### Animal models

All animal experiments were approved by the Institutional Animal Care and Use Committee of Tianjin University (TJUE-2020-174) and conformed to the Guide for the Care and Use of Laboratory Animals of the US National Institutes of Health (NIH Publication No. 85–23, updated 2011). To induce the HFpEF phenotype, male adult (6–8-week-old) C57BL/6J mice (Beijing HFK Bioscience Co. Ltd.) were administered N^ω^-Nitro-l-arginine methylester hydrochloride (L-NAME) (Sigma‒Aldrich, Germany) in sterile water (0.5 g/L; pH 7.4) and fed a 60% HFD as previously described [16]. For treatment, 10 mg/kg/day EMPA (MCE, USA) was administered via oral gavage for 28 days after 12 weeks of HFD+L-NAME treatment. At the end of the experiment, the mice were euthanized humanely through pentobarbital sodium (50 mg/kg, i.p.), and death was confirmed by exsanguination.

### Cell culture

H9C2 rat cardiomyoblasts (Procell CL-0089) were cultured in DMEM (Gibco, Thermo Fisher Scientific, USA) supplemented with 10% FBS (ExCell, Shanghai, China). To investigate the effect of EMPA on cell senescence, H9C2 cells were pretreated with EMPA (1 µM) for 30 min and then incubated with IFN-γ (50 µM) for 72 h. For STAT1 inhibition, cells at 60–70% confluence were transfected with STAT1 siRNA using a liposomal transfection reagent for 48 h (Yeasen, Shanghai, China).

### Echocardiography and doppler imaging

Animals were anesthetized and imaged using the VINNO 6 VET ultrasound system for small animals. Anesthesia was induced with 5% isoflurane and then reduced to 1–1.5% to maintain a heart rate in the range of 400–500 bpm. Cardiac systolic functions were measured using M-mode scanning. Pulsed-wave tissue Doppler of the mitral valve was used to measure diastolic function.

### Histopathological analysis

At the end of the experiment, the hearts were harvested and washed with cold phosphate-buffered saline (PBS). The collected hearts were fixed in 4% formaldehyde, embedded in paraffin, and cut into 5 μm-thick sections. Heart sections were stained with hematoxylin and eosin (HE) and Masson’s trichrome to evaluate the extent of cardiac hypertrophy and fibrosis, respectively.

### Immunohistochemistry

The sections were handled according to the manufacturer’s protocol (ZSJQ-BIO, Beijing, China). Briefly, paraffin-embedded sections were subjected to deparaffinization, hydration and antigen retrieval. H_2_O_2_ (3%) was then applied for 15 min to block endogenous peroxidase activity. Following blocking with 5% BSA at room temperature for 30 min, the sections were incubated with the following primary antibodies overnight at 4 °C: fibronectin (1:100, Abcam, Cambridge, USA) and p21 (1:100, Proteintech, Wuhan, China). The sections were incubated with HRP-conjugated secondary antibodies at room temperature for 30 min, visualized with a DAB Staining Kit (ZSJQ-BIO, Beijing, China), and finally counterstained with hematoxylin.

### ELISA

Plasma NT-proBNP and IFN-γ levels were assessed using an ELISA kit (Elabscience, Wuhan, China) according to the manufacturer’s protocol.

### Malondialdehyde (MDA) and SOD2 activity detection

Heart tissues were homogenized, and the levels of MDA and SOD2 activity (Beyotime, Shanghai, China) were measured according to the manufacturer’s instructions.

### Reactive oxygen species assay

After pretreatment with EMPA, H9C2 cells were treated with IFN-γ. Then the cells were labeled with 10 µmol/L DCFH-DA (Beyotime, Shanghai, China) for 20 min at 37 °C and then washed twice with PBS. Cell images were then captured using a fluorescence microscope (Nikon, Tokyo, Japan, Ex/Em = 488/525 nm).

### LDH release

LDH levels in plasma or cells were detected using an LDH activity assay kit (Solarbio, Shanghai, China). Briefly, the lysed cells were broken ultrasonically and centrifuged (8000×*g*, 10 min). Then, the supernatants were collected and mixed with the reaction mixture for 10 min. The absorbance was measured at 450 nm.

### Proteomic analysis

4D-DIA proteomic profiling was conducted in stored frozen heart tissues using the MetWare platform. Briefly, mouse heart samples were snap-frozen in liquid nitrogen. Protein extracts were obtained by homogenizing tissue in lysis buffer (7 M urea, 2 M thiourea, 4% SDS, 40 mM Tris–HCl, pH 8.5, 1 mM PMSF, 2 mM EDTA, 10 mM DTT) and then subjected to tryptic digestion. The tryptic peptide mixtures were subjected to nano LC–MS/MS on a timsTOF Pro mass spectrometer (Bruker). Qualitative and quantitative analyses of proteins were conducted with DIA-NN (v1.8.1) software. Differentially expressed proteins (DEPs) were defined as those with a cutoff of|log_2_(fold change)| greater than 0.26 and a *P* value less than 0.05.

### Western blot

Frozen myocardial tissue or cultured cells were lysed with ice-cold RIPA buffer containing protease and phosphatase inhibitors (Roche) and quantified with a BCA kit (Solarbio, Beijing, China). Protein samples (30–50 µg) were separated by SDS‒PAGE and transferred to a PVDF membrane (Millipore, Burlington, MA, USA). The membrane was blocked in 5% nonfat milk and incubated with the following primary antibodies overnight at 4 °C: GAPDH and α-tubulin (1:10,000, Proteintech, Wuhan, China), B-type natriuretic peptide (BNP, 1:1000, ABclonal), fibronectin and Bax (1:1000, Abcam), phospho-STAT1 (S727), STAT1, Ifit1, Ifit3, γH2AX, STING, IL6 and cGAS (1:1000, HUABIO), and SOD2, p16 and p21 (1:1000, Proteintech). After incubation with secondary antibodies for 1 h at room temperature, the signal intensities were visualized by a Supersignal chemiluminescence reagent (Yeasen, Shanghai, China). Relative protein levels were analyzed with ImageJ software.

### Quantitative real-time PCR

Total RNA was extracted with a Total RNA Purification Kit (Sangon, Shanghai, China) and reverse transcribed using a cDNA Synthesis Kit (Yeasen, Shanghai, China). Quantitative real-time PCR was performed with qPCR SYBR Green Mix (Yeasen, Shanghai, China) in a 7500 real-time PCR system (Life Technologies, USA). The relative mRNA expression levels were calculated by the 2^−ΔΔCT^ method, using glyceraldehyde-3-phosphate dehydrogenase (GAPDH) for normalization.

### Immunofluorescence

H9C2 cells were seeded on 4-well chamber slides for 24 h. After the indicated experimental treatments, the cells were fixed in 4% paraformaldehyde for 15 min and permeabilized with 0.2% Triton-X100 (Sigma Aldrich, St. Louis, MO, USA) for 10 min at room temperature. Nonspecific antigens were blocked with 3% BSA for 30 min, after which the membranes were incubated with primary antibodies overnight at 4 °C. After the cells were washed with PBS, they were incubated with fluorescein isothiocyanate (FITC)-conjugated secondary antibodies for 1 h at room temperature. Nuclei were counterstained with DAPI (ZSGB-BIO, Beijing, China). Images were acquired using a fluorescence microscope (Nikon, Tokyo, Japan).

### SA-β-gal staining

SA-β-gal activity (pH = 6.0) was detected using a commercial kit (Beyotime, Shanghai, China) as described previously [17]. Briefly, the cells were washed in PBS three times after treatment and fixed at room temperature for 15 min. SA-β-gal staining working solution was applied and the samples were incubated overnight at 37 °C. Images were acquired under a microscope (Nikon, Tokyo, Japan), and the percentage of SA-β-Gal-positive cells was calculated using ImageJ software.

### EdU assay

After treatment, the cells were cultured with medium containing 10 µM EdU (Beyotime, Shanghai, China) for 2 h. After EdU labeling, the cells were fixed with 4% paraformaldehyde and permeabilized with 0.3% Triton-X100 for 15 min at room temperature. Then, the cells were incubated with CLICK reaction buffer for 30 min and counterstained with Hoechst in the dark for 10 min. Images were acquired using a fluorescence microscope (Nikon, Tokyo, Japan).

### Lentivirus transfection

A lentivirus (LV3-H1-GFP-Puro) was constructed at GenePharma (Suzhou, China). Lentivirus transfection was performed according to the manufacturer’s instructions. H9C2 cells were transfected with lentivirus (1:10) and selected using 0.5 µg/mL puromycin for 3–7 days until all blank cells were killed. The surviving cells with green fluorescence were collected and subcultured in complete DMEM supplemented with 0.5 µg/mL puromycin.

### Statistical analysis

The data are expressed as the mean ± SEM. Differences were analyzed by unpaired Student’s t test, one-way ANOVA followed by Tukey’s post hoc test or two-way ANOVA followed by Bonferroni correction where appropriate. A *P* value < 0.05 was considered to indicate statistical significance. Statistical analyses were performed using GraphPad Prism software.

## Results

### EMPA ameliorates cardiac dysfunction in HFpEF mice

**Fig. 1:**
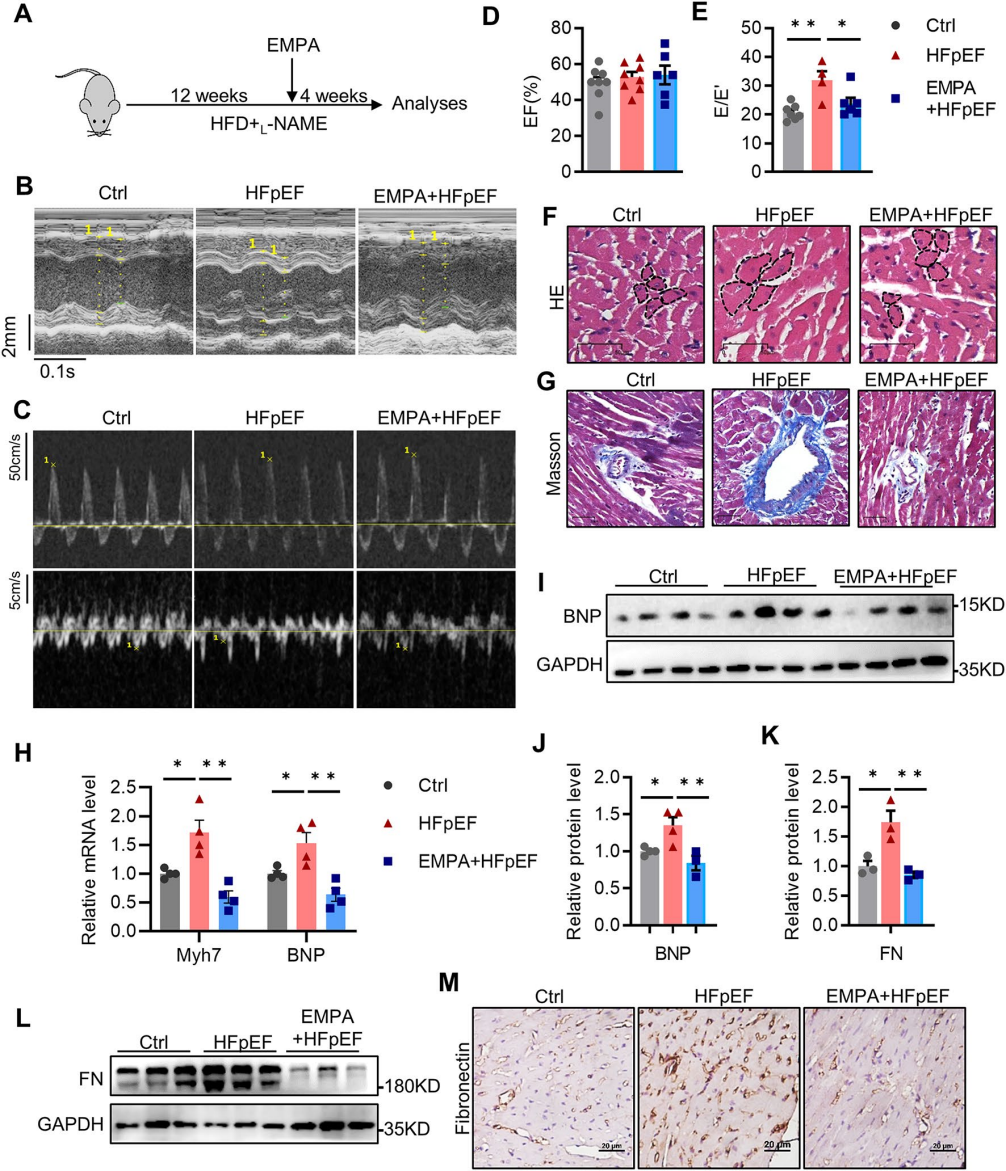
EMPA ameliorates cardiac dysfunction in HFpEF mice. **A** Flowchart of the animal experiments; **B** Representative images of left ventricular M-mode echocardiographic tracings; **C** Representative images of Doppler tracings; **D** Percentage of LVEF, *n* = 6–8; **E** Ratio between the mitral E wave and the E′ wave (E/E′), *n* = 4–7; **F**, **G** Representative images of hematoxylin and eosin and Masson trichrome staining, respectively; scale bars: 50 μm; **H** Relative cardiac mRNA levels of BNP and Myh7, *n* = 4; **I**, **J** Immunoblot analysis and quantification of BNP in the heart, *n* = 4; **K**, **L** Immunoblot analysis and quantification of fibronectin (FN) in the heart, *n* = 3; **M** Representative images of immunohistochemical staining of FN in heart tissues, scale bars: 20 μm. All the data are shown as the means ± SEM; one-way ANOVA with Tukey’s multiple comparisons test was used for comparisons; *P < 0.05, **P < 0.01

The flowchart of the animal experiments is shown in Fig. 1A. As expected, the mice exposed to the HFD+L-NAME regimen displayed a significant increase in body weight and NT-proBNP, a well-known diagnostic and prognostic biomarker for HF (Supplemental Fig. 1A, B). By echocardiography of the heart (Supplemental Fig. 1C), we observed an increase in the mitral E to E′-wave (E/E′) ratio, whereas the left ventricular ejection fraction (LVEF) remained preserved (Fig. 1B–E). These features suggested that the HFpEF model was successfully established. Compared with those in the HFpEF group, the E/E′ ratio and degree of obesity were lower in the EMPA-treated group, and there was no difference in the LVEF (Fig. 1B–E). In line with previous studies [18], the NT-proBNP level was reduced but not significantly after EMPA treatment (Supplemental Fig. 1B). These results indicated that EMPA improved cardiac diastolic dysfunction in HFpEF mice.

In addition, the cardiomyocyte size and fibrosis area were greater in the HFpEF mice than in the control mice, and these changes were impaired by EMPA treatment (Fig. 1F, G). Accordingly, EMPA strongly suppressed the increase in the mRNA levels of BNP and Myh7 (hypertrophy markers) in HFpEF hearts (Fig. 1H). In parallel, we observed an analogous reduction in BNP protein in EMPA-treated hearts compared with that in HFpEF hearts (Fig. 1I, J). Similarly, the HFpEF hearts exhibited increased levels of the fibronectin protein, which were decreased by EMPA treatment (Fig. 1K–M). Taken together, these data indicated that EMPA alleviated cardiac hypertrophy and fibrosis in HFpEF mice, ultimately improving diastolic dysfunction.

### Effects of EMPA on the protein expression profile of HFpEF mice

**Fig. 2:**
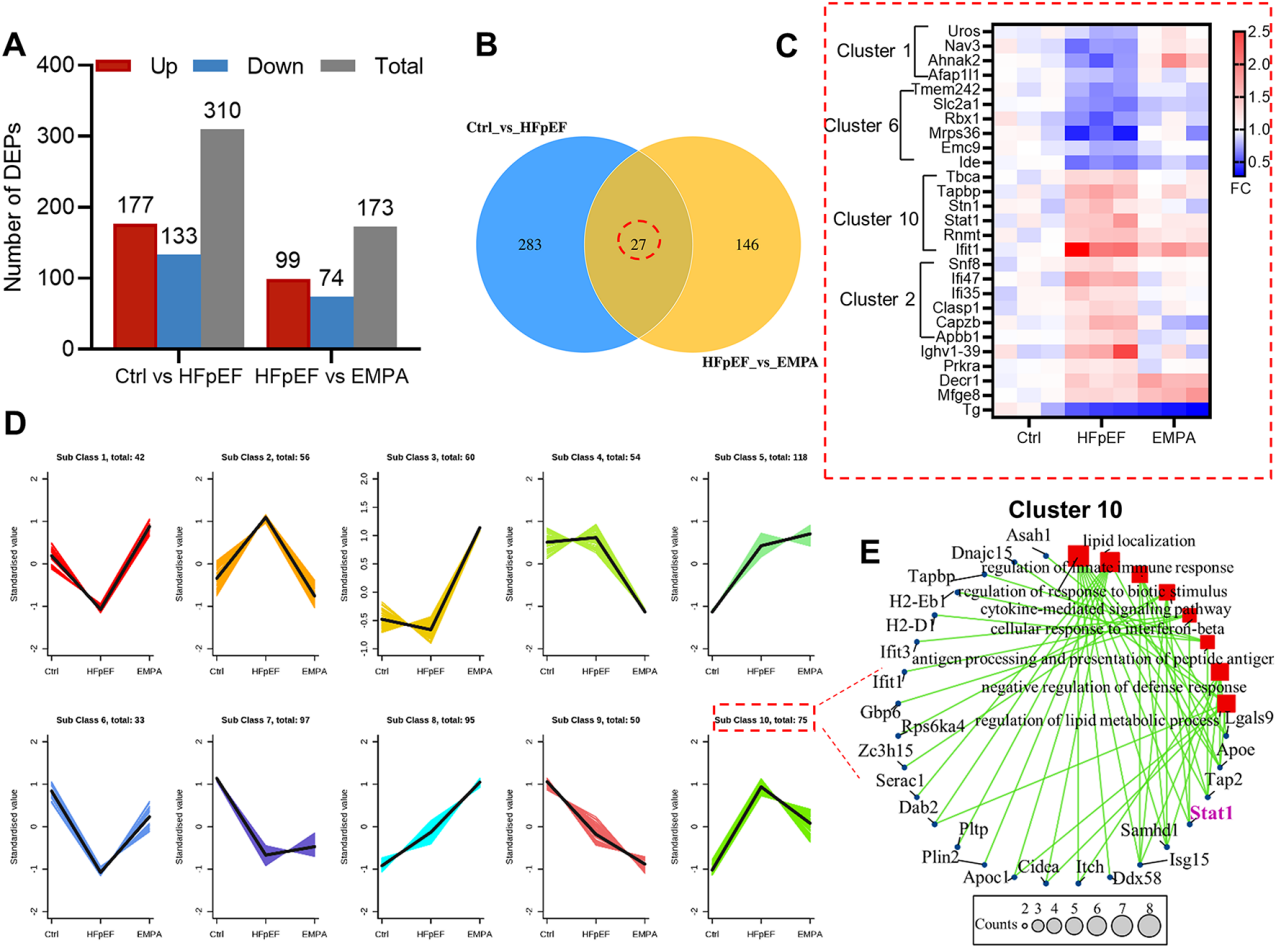
Effects of EMPA on the protein expression profile of HFpEF mice. 4D-DIA quantitative proteomics analysis was performed at the heart tissue level in control, HFpEF and EMPA-treated mice. **A** The number of DEPs that were upregulated, downregulated and in total; **B** Venn diagram of DEPs for the three groups; **C** Heatmap of coregulated proteins in all groups; **D** K-means clusters containing ten subclasses; **E** Gene Ontology (GO) enrichment and interaction network analysis based on DEPs in cluster 10

To understand the potential mechanisms by which EMPA protects against cardiac deterioration, we performed 4D-DIA-based quantitative proteomic analysis of heart tissue. Principal component (PC) analysis revealed separable clusters among the three groups (Supplemental Fig. 2A). In total, we identified 5702 proteins that were then used for comparative analysis and defined differentially expressed proteins (DEPs) as those with a cutoff of|log_2_(fold change)| greater than 0.26 and a *P* value less than 0.05 (Supplemental Fig. 2B). Among them, 310 DEPs (177 upregulated and 133 downregulated) were identified in HFpEF hearts compared with control hearts. We further analyzed the target proteins regulated by EMPA and identified 173 DEPs (99 upregulated and 74 downregulated) between the HFpEF and EMPA groups (Fig. 2A and Supplemental Table 1). Furthermore, Venn diagrams showed 27 coregulated proteins in all groups (Fig. 2B). The decreased expression of glucose transporter 1 (GLUT1, also called Slc2a1) in HFpEF mice could be restored by EMPA treatment (Fig. 2C and Supplemental Fig. 3). This finding is consistent with previous studies showing that EMPA enhances GLUT1 expression, which is generally lower in HFpEF patients [19, 20]. Additionally, we observed a similar trend in the expression of mitochondrial respiration (Tmem242), E3 ubiquitin ligase (Rbx1), neuron navigator 3 (Nav3), and nucleoprotein (Ahnak2). In contrast, the expression patterns of interferon response genes (STAT1, Ifit1, Ifi35 and Ifi47), an antigen-processing molecule (Tapbp) and a mitochondrial protein (Decr1) were opposite (Fig. 2C). Moreover, K-means cluster analysis revealed that all DEPs were classified into ten types based on their expression patterns (Fig. 2D). Most coregulated proteins could be found in clusters 1, 2, 6 and 10 with a U-shaped or reverse U-shaped profile (Fig. 2C, D). Further gene ontology (GO) enrichment and interaction network analysis of the DEPs in cluster 10 revealed a potential connection between lipid metabolism and the innate immune response (Fig. 2E).

### STAT1 is the hub transcription factor involved in HFpEF pathogenesis

**Fig. 3:**
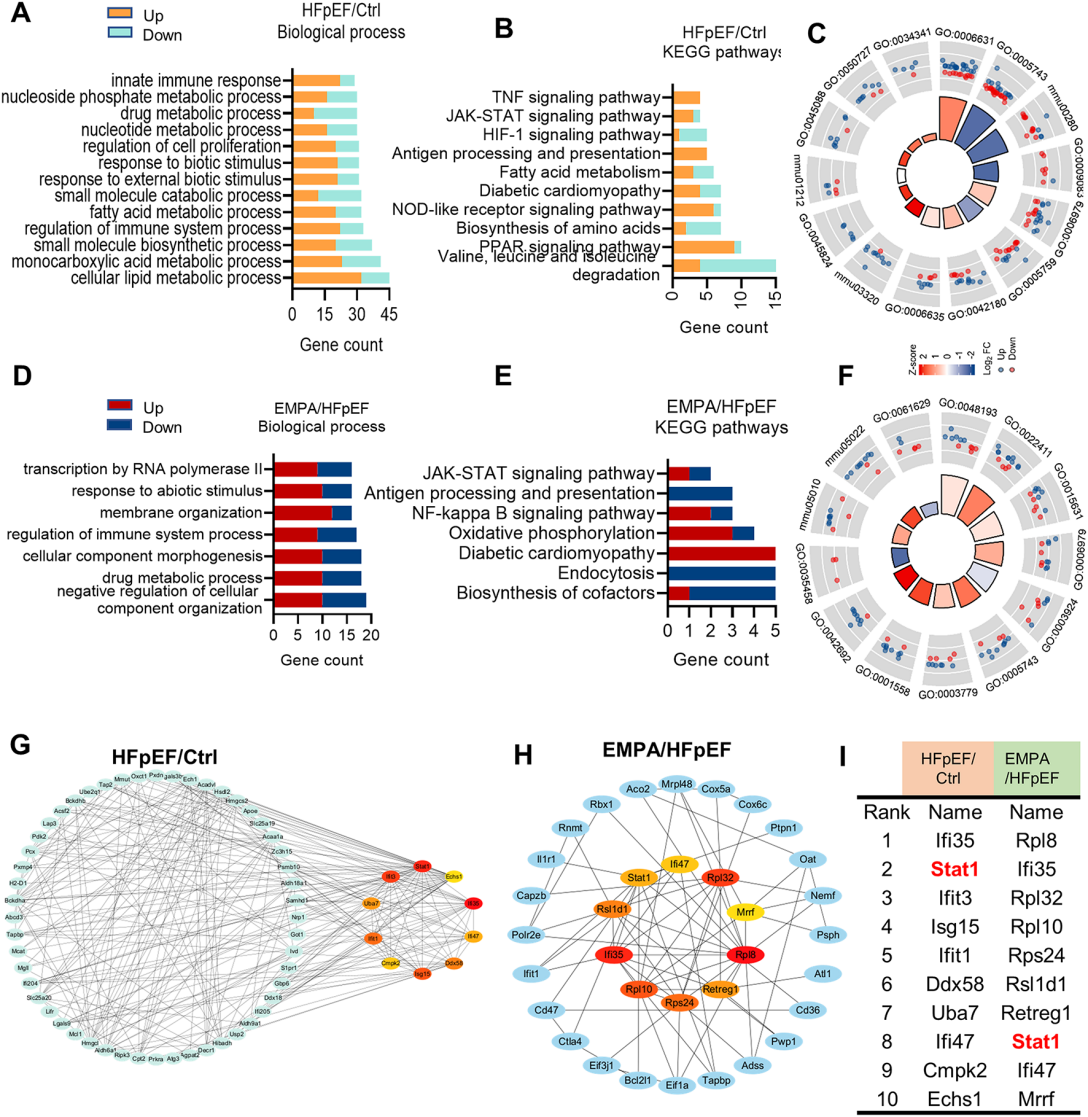
STAT1 is the hub transcription factor involved in HFpEF pathogenesis. **A**, **B** Enrichment analysis of gene ontology (GO)–biological process and KEGG pathway between the control and HFpEF groups; the orange or green color represents upregulated or downregulated, respectively; **C** The interaction network diagram between the enriched gene and the functional pathway set based on DEPs in HFpEF mice; **D**, **E** GO enrichment and KEGG pathway identification of DEPs between HFpEF and EMPA group; the red or blue color represents upregulated or downregulated, respectively; **F** The interaction network diagram between the enriched gene and the functional pathway set based on DEPs in EMPA mice; **G**, **H** Top 10 hub genes based on DEPs in the HFpEF/Ctrl and EMPA/HFpEF group with Cytoscape software; **I** The table shows the top 10 hub genes ranked by the MCC method

To explore the key factors contributing to the pathogenesis of HFpEF, we performed enrichment analysis. GO analysis revealed that the enriched biological processes in HFpEF hearts were mainly related to (I) cellular lipid metabolic processes, (II) regulation of immune system processes, (III) regulation of cell proliferation, and (IV) the innate immune response (Fig. 3A). Moreover, the enriched pathways involved valine, leucine and isoleucine degradation; the PPAR signaling pathway; the NOD-like receptor signaling pathway; and the JAK-STAT signaling pathway (Fig. 3B). These results revealed obvious changes in myocardial lipid and branched-chain amino acid metabolism, which are consistent with the cardiometabolic features observed in this model [16]. The interaction network diagram further indicated that the innate immune response (GO:0045824) was significantly enhanced in the HFpEF group (Fig. 3C). Moreover, pathways associated with the regulation of immune system processes, mainly the JAK-STAT1 signaling pathway and antigen processing and presentation, were also enriched in EMPA-treated hearts (Fig. 3D, E). Interaction network analysis demonstrated that EMPA treatment upregulated the biological processes of cell growth (GO:0001558) and differentiation (GO:0042692) but downregulated the cellular response to interferon-beta (GO:0035458) (Fig. 3F). Importantly, among the hub genes, STAT1 was the only transcription factor strongly associated with the interferon response (Fig. 3G and Supplementary Table 1). In comparison with other proteomic results in hypertension-associated HFpEF mice [21], we identified 49 coregulated proteins (Supplemental Fig. 4A) and found that biological processes, such as the fatty acid metabolic process and response to type I interferon, were enriched in the two models (Supplemental Fig. 4B, C). Interestingly, STAT1 was also significantly upregulated in hypertension-associated HFpEF mice (Supplemental Fig. 4D). These results confirmed that STAT1 is a key transcription factor contributing to pathological changes in HFpEF. Overall, we speculated that EMPA could protect against cardiac deterioration during HFpEF partly by reducing the immune response through the regulation of STAT1 activity.

### EMPA inhibits the cardiac inflammatory response and aging in HFpEF mice

**Fig. 4:**
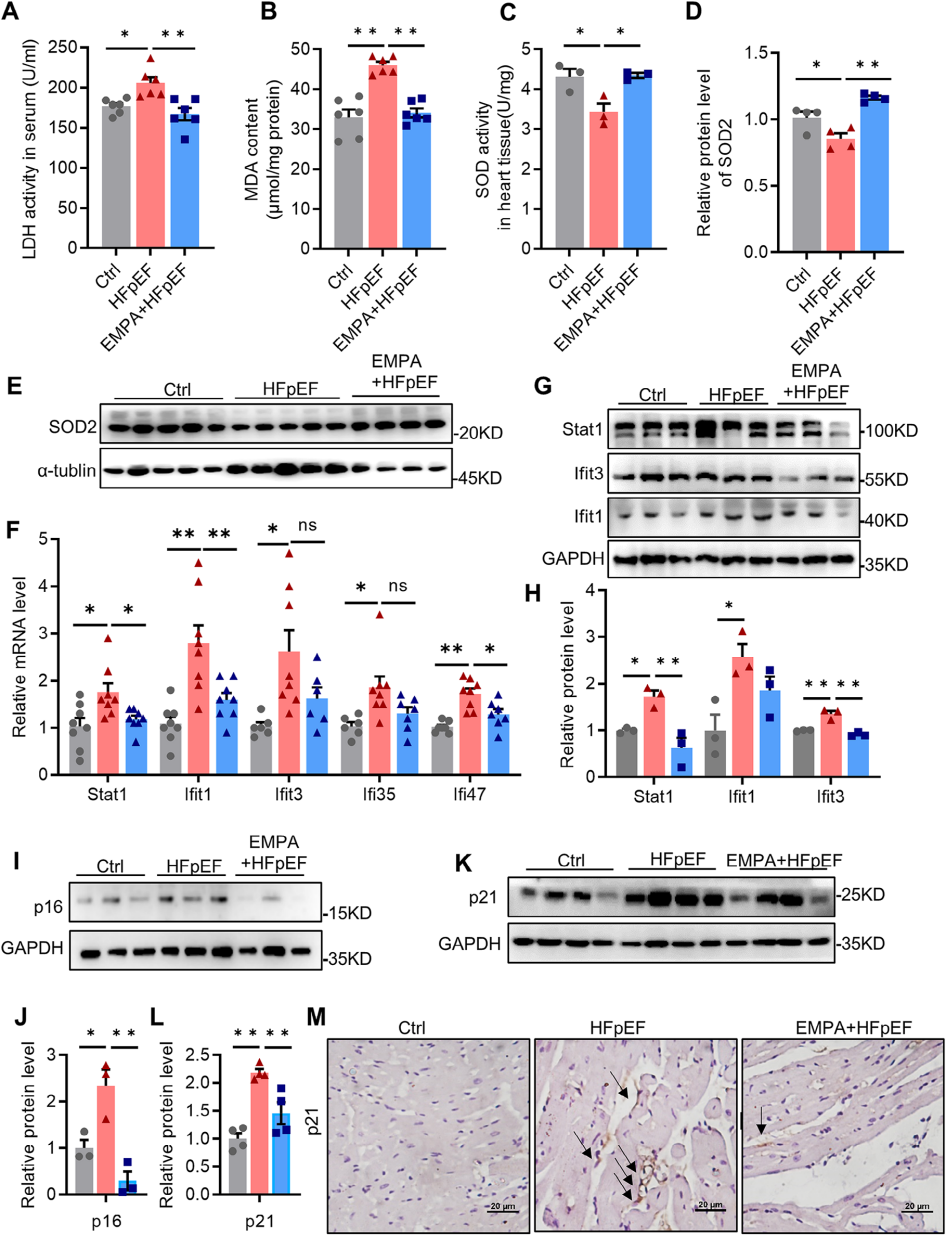
EMPA inhibits the cardiac inflammatory response and aging in HFpEF mice. **A** The serum levels of LDH activity in different groups, *n* = 6; **B**, **C** The levels of MDA content (*n* = 6) and SOD activity (*n* = 3) in heart tissues; **D**, **E** Immunoblot analysis and quantification of SOD2 protein, *n* = 4; **F** The mRNA expression of Stat1, Ifit1, Ifit3, Ifi35 and Ifi47 in the heart, *n* = 6–8; **G**, **H** Representative western blot image and quantification of Stat1, Ifit1 and Ifit3 in the heart, *n* = 3; **I**, **J** Representative immunoblot images and quantification of p16 in the heart, *n* = 3; **K**, **L** Immunoblot analysis and relative expression of p21 in the heart, *n* = 4; **M** Representative images of immunohistochemical staining of p21, scale bars:20 μm. All the data are shown as the means ± SEM; one-way ANOVA with Tukey’s multiple comparisons test was used for comparisons; *P < 0.05, **P < 0.01

Lactate dehydrogenase (LDH) is a marker of myocardial injury. We found that LDH activity in plasma was significantly elevated in HFpEF mice compared with controls. However, EMPA treatment strongly restrained LDH release (Fig. 4A). Malondialdehyde (MDA) levels reflect the extent of oxidative stress, particularly lipid peroxidation [22]. The results showed that the HFD+L-NAME regimen resulted in a significant increase in the MDA level in the heart, while EMPA administration suppressed this increase (Fig. 4B). In contrast, SOD activity was inhibited in HFpEF hearts but restored by EMPA treatment (Fig. 4C). Similar changes in the SOD2 protein level were observed in the heart (Fig. 4D, E). Combined with the proteomic results, we next detected whether changes related to interferon signaling contributed to the protective mechanism of EMPA during the pathological progression of HFpEF. We confirmed that the mRNA levels of IFN response genes, including STAT1, Ifit1, Ifit3, Ifi35 and Ifi47, were increased in HFpEF hearts but decreased in EMPA-treated hearts (Fig. 4F). Similarly, the significantly elevated protein levels of STAT1, Ifit1 and Ifit3 were inhibited after EMPA administration (Fig. 4G, H). These results indicate that EMPA alleviates cardiac injury by inhibiting the inflammatory response, especially signaling related to the IFN response.

Chronic inflammation is a risk factor for aging [11]. As indicated by Fig. 4I–L, the protein levels of both p21 and p16 (senescence markers) increased more than 2.0-fold in HFpEF hearts compared with those in controls, while the EMPA-treated hearts exhibited a significant decrease in these levels. Consistently, the immunohistochemistry results confirmed that EMPA treatment suppressed the increase in p21 protein expression in HFpEF hearts (Fig. 4M). Taken together, these findings suggest that EMPA inhibits the cardiac inflammatory response and aging in HFpEF mice.

### Stat1 activation downstream of IFN-γ mediated cardiomyocyte senescence

**Fig. 5:**
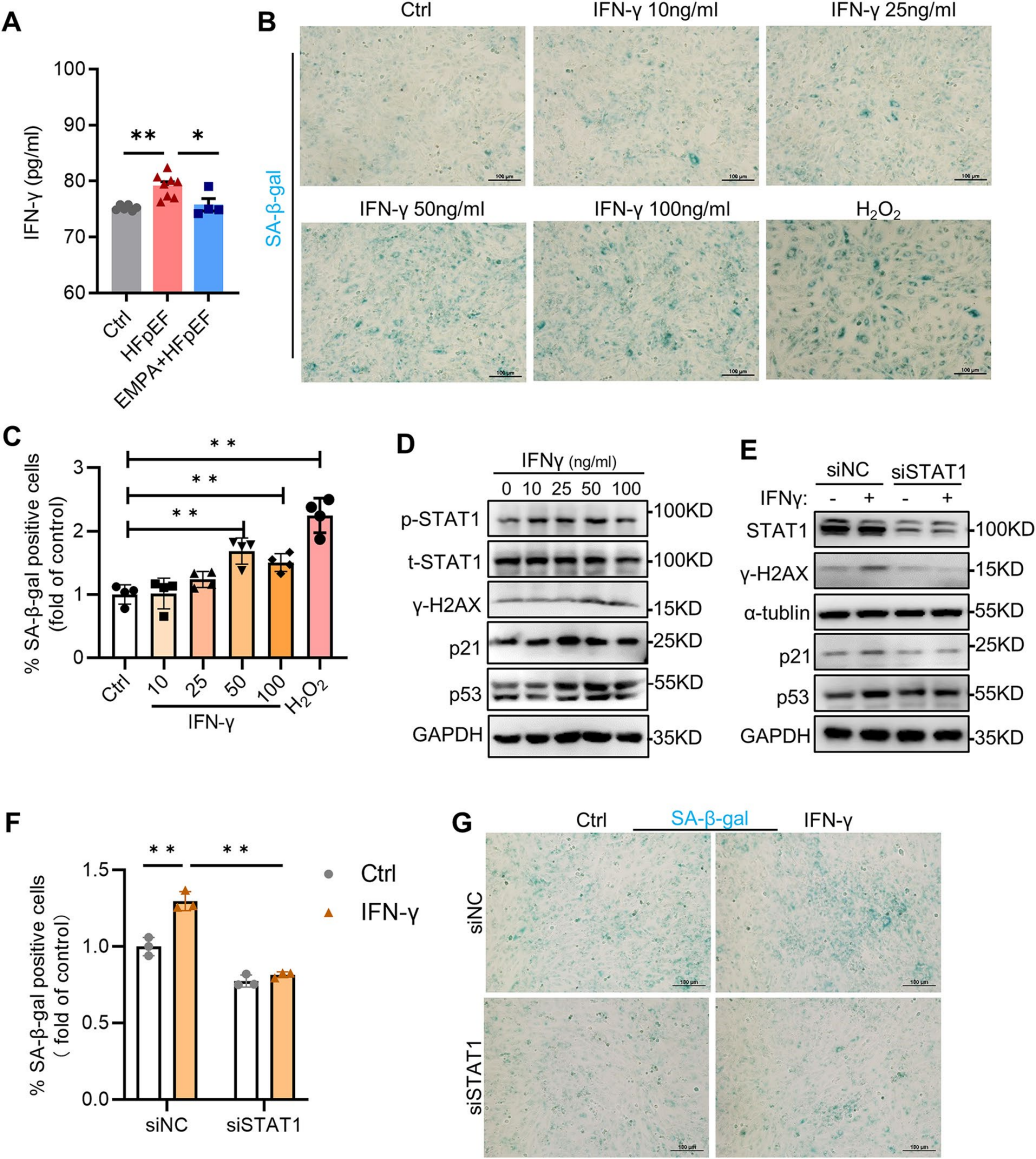
Stat1 activation downstream of IFN-γ mediated cardiomyocyte senescence. **A** The serum levels of IFN-γ in different groups, *n* = 4–8; **B** Representative images of SA-β-gal staining in H9C2 cells exposed to IFN-γ at different concentrations for 72 h or normal culture after 200 µM H_2_O_2_ treatment for 2 h, scale bars: 100 μm; **C** Quantification of SA-β-gal-positive cells in different groups, *n* = 4; **D** Representative immunoblot images of p-STAT1, t-STAT1 and γ-H2AX in H9C2 cells exposed to IFN-γ at different concentrations; **E** Immunoblot analysis for STAT1 and γ-H2AX in H9C2 cells after STAT1 inhibition; **F**, **G** Quantification and representative images of SA-β-gal staining in H9C2 cells exposed to 50 µM IFN-γ after transfection with STAT1 siRNA, *n* = 3; scale bars:100 μm. All the data are shown as the means ± SEM; one-way ANOVA with Tukey’s multiple comparisons test was used for comparisons; *P < 0.05, **P < 0.01

To confirm how EMPA improves heart function by regulating IFN signaling, IFN-γ levels were first detected. Our results showed that the HFpEF mice had a slightly greater IFN-γ level, which was restored to the levels of the control mice by EMPA treatment (Fig. 5A). Myocardial IFN-γ signaling increases with age, and genes downstream of IFN-γ signaling, including *Irf1*, *Irf9*, *Jak1*, *Stat1* and other IFN-γ response genes, are upregulated in aged hearts [23]. We next investigated the role of IFN-γ in CM senescence. We applied different concentrations of IFN-γ (10, 20, 50 and 100 ng/ml) for three days to H9C2 cells and used H_2_O_2_ treatment (200 µM for 2 h) as a positive control. The results of SA-β-gal staining showed that cellular senescence significantly increased in an IFN-γ concentration-dependent manner (Fig. 5B-C). This finding was corroborated by immunoblotting for senescence-associated p53, p21 and γH2AX (a marker of double-strand DNA damage). These protein levels also increased in an IFN-γ concentration-dependent manner (Fig. 5D). STAT1 phosphorylation at the S727 locus is required for STAT1 transcriptional and biological activity in response to IFNγ [24]. As shown in Fig. 5D, IFN-γ also caused concentration-dependent STAT1 activation. To investigate the potential role of STAT1 in IFN-γ-induced cellular senescence, we treated cells with STAT1 siRNA to knock down its expression. Compared with IFN-γ-treated cells, STAT1-silenced CMs showed decreased levels of p53, p21 and γH2AX (Fig. 5E) and SA-β-gal activity (Fig. 5F–G). These data indicate that STAT1 activation mediates IFN-γ-induced CM senescence.

### EMPA inhibits IFN-γ-induced cardiomyocyte senescence by blocking STAT1 activation

**Fig. 6:**
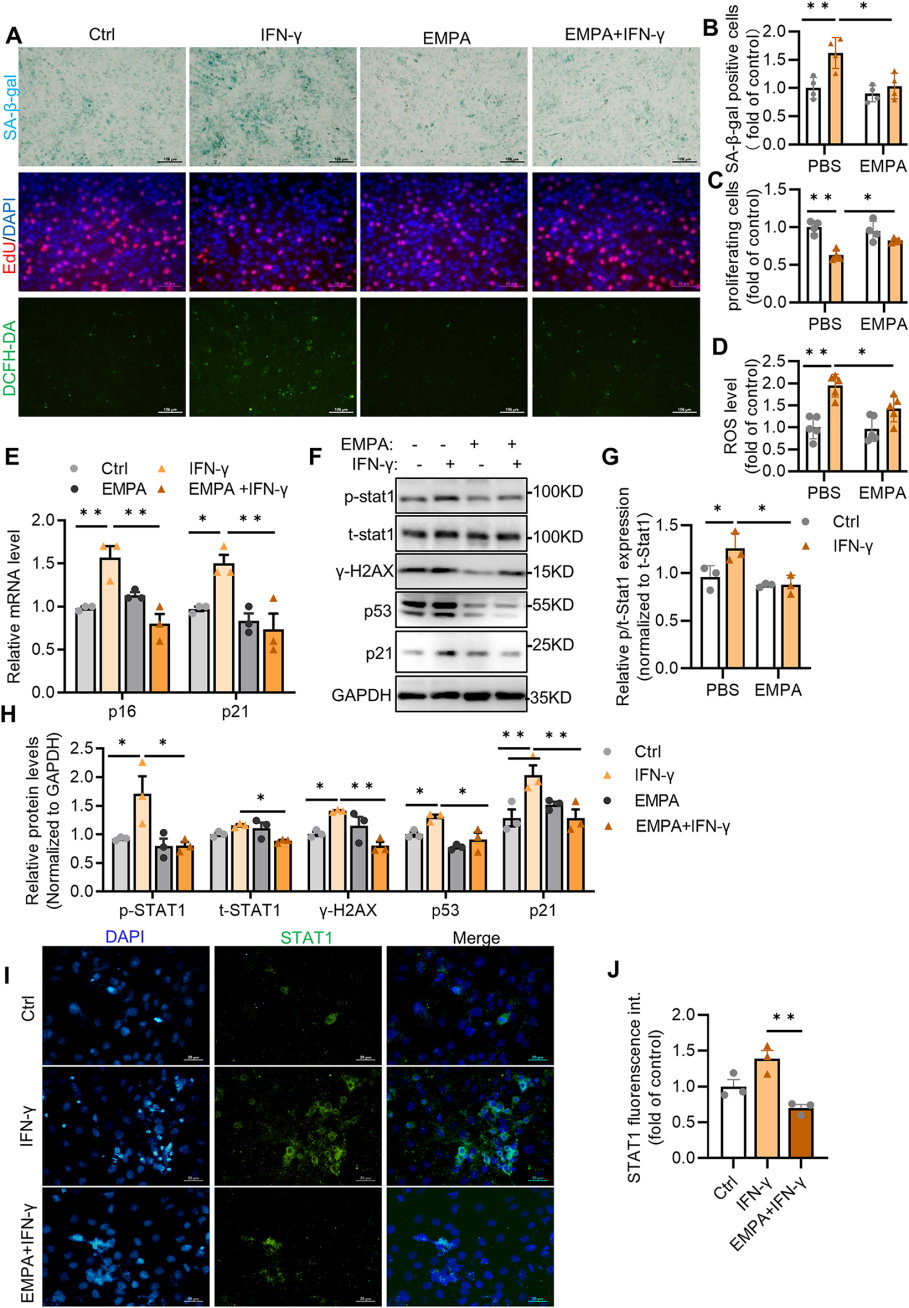
EMPA inhibits IFN-γ-induced cardiomyocyte senescence by blocking STAT1 activation. H9C2 cells were pretreated with 1 µM EMPA and then treated with 50 µM IFN-γ. **A** Representative images of SA-β-gal staining (top), EdU staining (middle) and DCFH-DA staining (bottom); **B** Quantification of SA-β-gal-positive cells, *n* = 4; **C** Quantification of proliferating cells, *n* = 4; **D** Quantification of ROS levels in H9C2 cells, *n* = 5; **E** mRNA expression of p16 and p21 in H9C2 cells, *n* = 3; **F–H** Immunoblot analysis and quantification of p-STAT1, t-STAT1 and γ-H2AX in H9C2 cells, *n* = 3. **I**, **J** Representative images and quantification of anti-STAT1 staining under the indicated conditions, *n* = 3. Scale bar: 20 μm. All the data are shown as the means ± SEM; one or two-way ANOVA followed by Bonferroni correction was used for comparisons; *P < 0.05, **P < 0.01

To explore the effect of empagliflozin on cell senescence, H9C2 cells were pretreated with 1 µM EMPA. Oxidative stress contributes to cell senescence, along with cell cycle arrest. In addition to increased SA-β-gal activity, we also found that IFN-γ-treated cells exhibited significantly increased oxidative stress but decreased proliferation. However, EMPA treatment markedly delayed CM senescence and oxidative stress caused by IFN-γ and promoted cell proliferation (Fig. 6A–D). The same results were observed in cells after H_2_O_2_ treatment (Supplemental Fig. 5A). Additionally, EMPA treatment significantly inhibited the cell injury induced by IFN-γ (Supplemental Fig. 5B). The results of quantitative real-time PCR showed that the increase in the mRNA levels of p16 and p21 in IFN-γ treated cells was strongly suppressed by EMPA (Fig. 6E). In addition, the increase in STAT1 activity and the expression of senescence markers p53, p21 and γ-H2AX induced by IFN-γ was significantly inhibited by EMPA treatment (Fig. 6F–H). The immunofluorescence results confirmed the inhibitory effect of EMPA on STAT1 activation in response to IFN-γ treatment (Fig. 6I–J). Thus, we speculate that EMPA suppresses IFN-γ-induced CM senescence by inhibiting STAT1 activation.

### The transcription factor STAT1 positively regulates STING expression

**Fig. 7:**
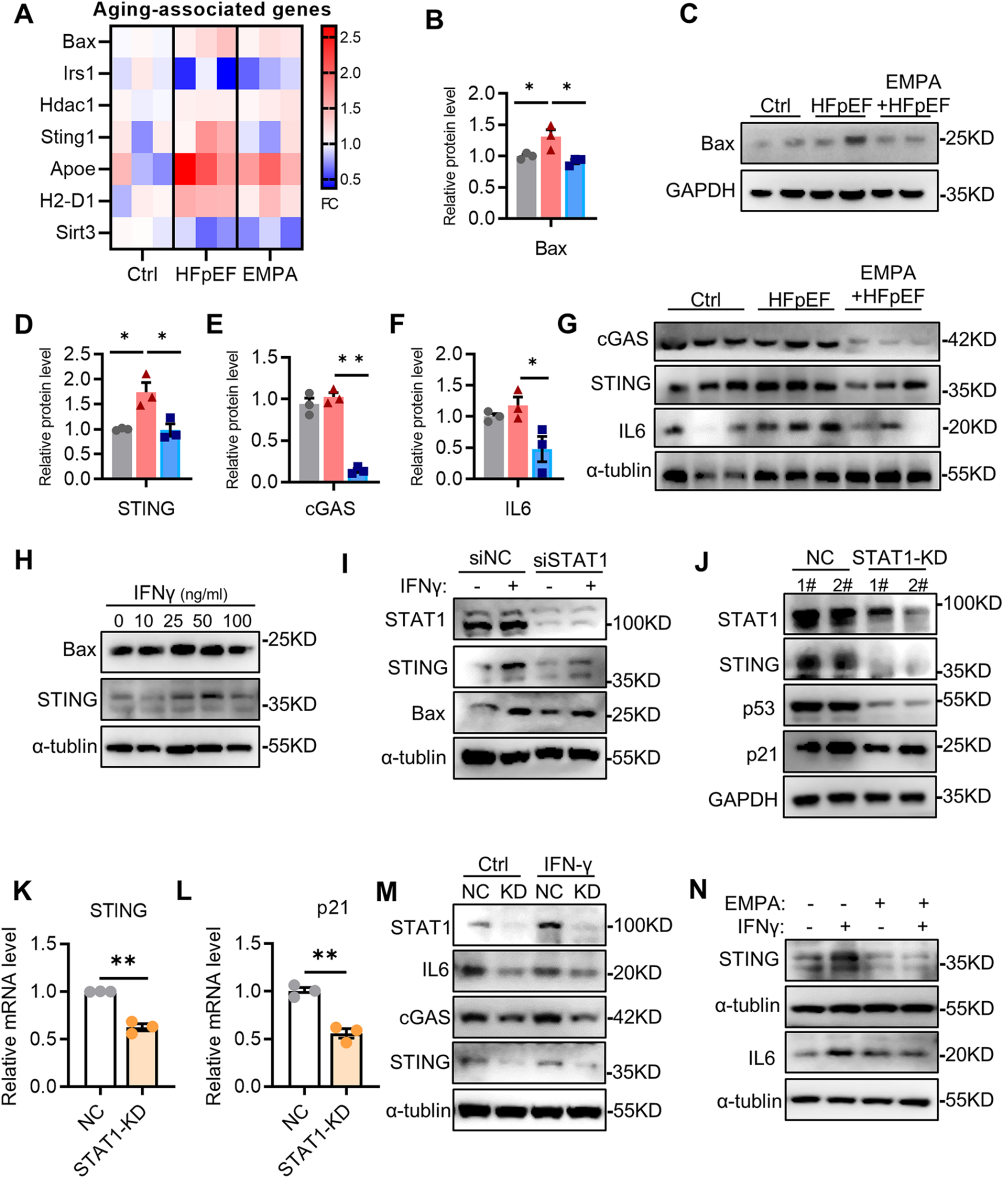
The transcription factor STAT1 positively regulates STING expression. **A** Heatmap of aging-associated genes identified in hearts from Ctrl, HFpEF and EMPA-treated mice by proteomics analysis; **B**, **C** Immunoblot analysis and quantification of Bax protein in heart tissues, *n* = 3; **D–G** Quantification and immunoblot analysis of cGAS, STING and IL6 protein in heart tissues, *n* = 3; **H** Representative immunoblot images of Bax and STING in H9C2 cells exposed to IFN-γ at different concentrations; **I** Representative immunoblot images of STAT1, STING and Bax protein in H9C2 cells treated with IFN-γ after transfection with STAT1 siRNA; **J** Representative immunoblot images of STAT1, STING, p53 and p21 protein in STAT1-knockdown (KD) cells; **K**, **L** mRNA levels of STING and p21 in control and STAT1-KD cells, *n* = 3; **M** Representative immunoblot images of STAT1, cGAS, STING and IL6 protein in STAT1-KD cells treated with IFN-γ; **N** Representative immunoblot images of STING and IL6 protein in H9C2 cells treated with IFN-γ and EMPA pretreatment. All the data are shown as the means ± SEM; one-way ANOVA with Tukey’s multiple comparisons test or unpaired Student’s t-test was used for comparisons; *P < 0.05, **P < 0.01

We next analyzed the potential targets regulated by the STAT1 transcription factor. Proteomic analysis of aging-associated genes revealed that the increased expression of the Bax, Sting, ApoE, and H2-D1 genes in HFpEF hearts was inhibited by EMPA treatment in mice. However, the Irs1 and Sirt3 genes exhibited opposite expression trends (Fig. 7A). Immunoblotting further confirmed that the protein levels of Bax, cGAS, STING and IL6 were greater in the HFpEF mice than in the control mice and that these changes were attenuated in the EMPA-treated mice (Fig. 7B–G). JAK2-STAT1 activates Bax expression to induce mitochondrial dysfunction and cell death [25]. We next examined whether STAT1 regulated Bax expression. We observed that IFN-γ increased Bax expression in a concentration-dependent manner. Similarly, STING expression was also enhanced in response to IFN-γ treatment (Fig. 7H). However, STAT1 inhibition d ecreased the protein expression of Bax and STING (Fig. 7I). A recent study from *Victorelli* reported that increased Bax expression in senescent cells causes the release of mitochondrial DNA, which in turn activates the cGAS–STING pathway, a major regulator of the SASP [26]. Notably, the cGAS–STING pathway is an important driver of aging-related inflammation [27]. To further study whether STAT1 regulates STING expression during aging, a stable cell line with STAT1 knockdown (KD) was established using a recombinant lentivirus system (Supplemental Fig. 6). Consistent with the results of STAT1 inhibition by siRNA, STING expression was reduced in STAT1-KD cells compared with that in normal control cells and the senescence markers p53 and p21 were also eliminated by STAT1 knockdown (Fig. 7J). Real-time PCR showed that knocking down STAT1 resulted in a significant decrease in the Sting mRNA level, accompanied by an obvious reduction in the p21 mRNA level (Fig. 7K, L). After IFN-γ treatment, the increases in the levels of the cGAS, STING and IL6 proteins were inhibited in STAT1-KD cells (Fig. 7M). Importantly, similar to STAT1 inhibition, EMPA treatment also reduced the increase in STING and IL6 expression caused by IFN-γ (Fig. 7N and Supplemental Fig. 7). Therefore, STAT1/STING axis likely promotes IFN-γ-induced CM senescence.

## Discussion

The mechanism by which SGLT2i protects the heart can be attributed to its multitarget effects on myocardial energy metabolism, inflammation, autophagy, and cardiac remodeling [7]. However, the underlying mechanism of EMPA in HFpEF patients remains unclear. In this work, we observed that EMPA administration effectively improved heart function in HFpEF mice fed a HFD+L-NAME. Moreover, we described the protein expression profiles of the hearts of HFpEF mice treated with or without EMPA. Further bioinformatics analysis demonstrated the involvement of the immune response, especially the JAK-STAT1 signaling pathway, during HFpEF. Notably, the coregulated protein STAT1, a key downstream factor of the IFN-γ response, was significantly upregulated in HFpEF hearts, whereas it was downregulated in EMPA-treated hearts. In addition, its activation was observed in IFN-γ-induced CM senescence in vitro. Interestingly, we found that STAT1 positively regulated STING expression, which may exacerbate the SASP and cell senescence. Taken together, these findings suggest that EMPA can mitigate cardiac inflammation and aging during HFpEF by blocking the STAT1–STING axis.

Our results revealed that the innate immune response and aging process are enhanced in HFpEF mice. Aging has long been hypothesized to be the major contributor to HFpEF. Chronic inflammation, which is accompanied by cellular senescence, immunosenescence, organ dysfunction, and age-related diseases, appears to be closely linked to aging [11]. The molecular connections between senescence and inflammation in HFpEF are complex. Recent studies have demonstrated that IFN-γ and TNF-α synergistically promote STAT activation and augment inflammation, ultimately aggravating cellular senescence in endothelial cells [28]. However, EMPA can directly target human CMs by inhibiting CXCL10 secretion induced by IFN-γ+TNFα treatment in association with Stat1 pathway impairment [29]. Richard et al. combined IFN-γ, IL-1β, and poly(I: C) to mimic the inflammatory cytokine storm induced by SARS-CoV-2 infection in cardiac organoids and reported that STAT1 is the transcription factor most strongly regulated during cytokine storm induced diastolic dysfunction [24]. IFN-γ, a primary agonist of STAT1 phosphorylation, has been identified as the dominant factor that causes diastolic dysfunction [24]. Thus, we used IFN-γ as a stimulator to investigate the impact of STAT1 on CM function in vitro. H9C2 cells exposed to IFN-γ displayed a senescent phenotype; however, cell senescence was inhibited by STAT1 silencing. Consistently, the IFNγ-Stat1 axis drives aging-associated loss of intestinal tissue homeostasis, and blocking IFN-γ signaling reverses the aging phenotype [30].

Interestingly, STAT1 was also significantly upregulated in hypertension-associated HFpEF mice. These results confirmed that STAT1 is a key transcription factor during pathological changes in HFpEF and may be linked to inflammation and senescence in CMs. Therefore, the use of anti-inflammatory drugs targeting the IFNγ-Stat1 axis is a promising strategy for treating aging and age-related diseases.

The cGAS–STING pathway has emerged as a key mediator of inflammation during infection, cellular stress and tissue damage [31]. Recent studies have shown that this pathway also plays an important role in the pathophysiology of cardiovascular diseases. For example, the cGAS–STING pathway promoted doxorubicin-induced cardiotoxicity [32]. A recent study revealed that cGAS–STING drives aging-related inflammation and neurodegeneration, while its inhibitor (H-151) inhibited the inflammatory response and improved cognition by inhibiting the SASP [27]. Consistent with previous observations that the cGAS–STING pathway functions downstream of IFNγ-STAT1 signaling [33], STING expression is downregulated following STAT1 deletion in senescent preadipocytes [34]. Here, we showed that cGAS and STING expression in CMs was reduced by STAT1 inhibition. In fact, a STAT1 binding site was identified in the STING promoter [35]. These results suggest that STAT1 activation may promote cellular senescence by regulating STING expression. Therefore, we speculate that the cGAS–STING pathway, a downstream signaling pathway of STAT1 activation, regulates cell senescence and aging. Given that senescent cells release DNA into the cytoplasm, does STAT1 directly regulate STING transcriptionally or indirectly by affecting extracellular DNA? Conversely, others have reported a negative role of STING in regulating the activation of JAK1-STAT1 signaling triggered by cytosolic DNA [36]. Future studies should focus on the functional mode and potential mechanism of the STAT1–STING axis in the cardiovascular system. In addition, we are very interested in the role of the STAT1–STING axis in CM metabolism because metabolic reprogramming, particularly lipid metabolism, was observed in HFpEF hearts in our study.

Anti-inflammatory therapies show promise for treating HF. In addition to reducing the inflammatory response, SGLT2i may have cardioprotective effects on HF for multiple reasons, such as cellular energy metabolism, mitochondrial function and oxidative stress [37]. The protective effects on DNA methylation and metabolites associated with cellular stress have also been reported [38, 39]. Recently, increasing evidence has suggested that SGLT2is, which induce a fasting-like metabolic paradigm [40], can also be repurposed as anti-aging drugs [12]. In the present study, we demonstrated that EMPA reduced the senescence markers p16 and p21 in HFpEF hearts. Furthermore, EMPA administration attenuated IFN-γ-induced oxidative stress and senescence in CMs by inhibiting STAT1 activation in vitro. It has been reported that the innate immune system receptor/interferon/Stat1 axis triggers inflammaging and that the expression of STAT1, the master TF mediating the transcriptional response to inflammatory cascade inactivity, increases during aging but decreases after dietary restriction [41]. Previous studies have shown that EMPA significantly reduces proinflammatory factor levels and inhibits STAT1 activation in a dose-dependent manner [42]. Cardiomyocytes do not express SGLT2. Thus, the beneficial effects of EMPA on cardiomyocytes must be indirect, and further experiments are required to fully understand the anti-aging mechanism of EMPA and other SGLT2is.

## Conclusions

**Fig. 8:**
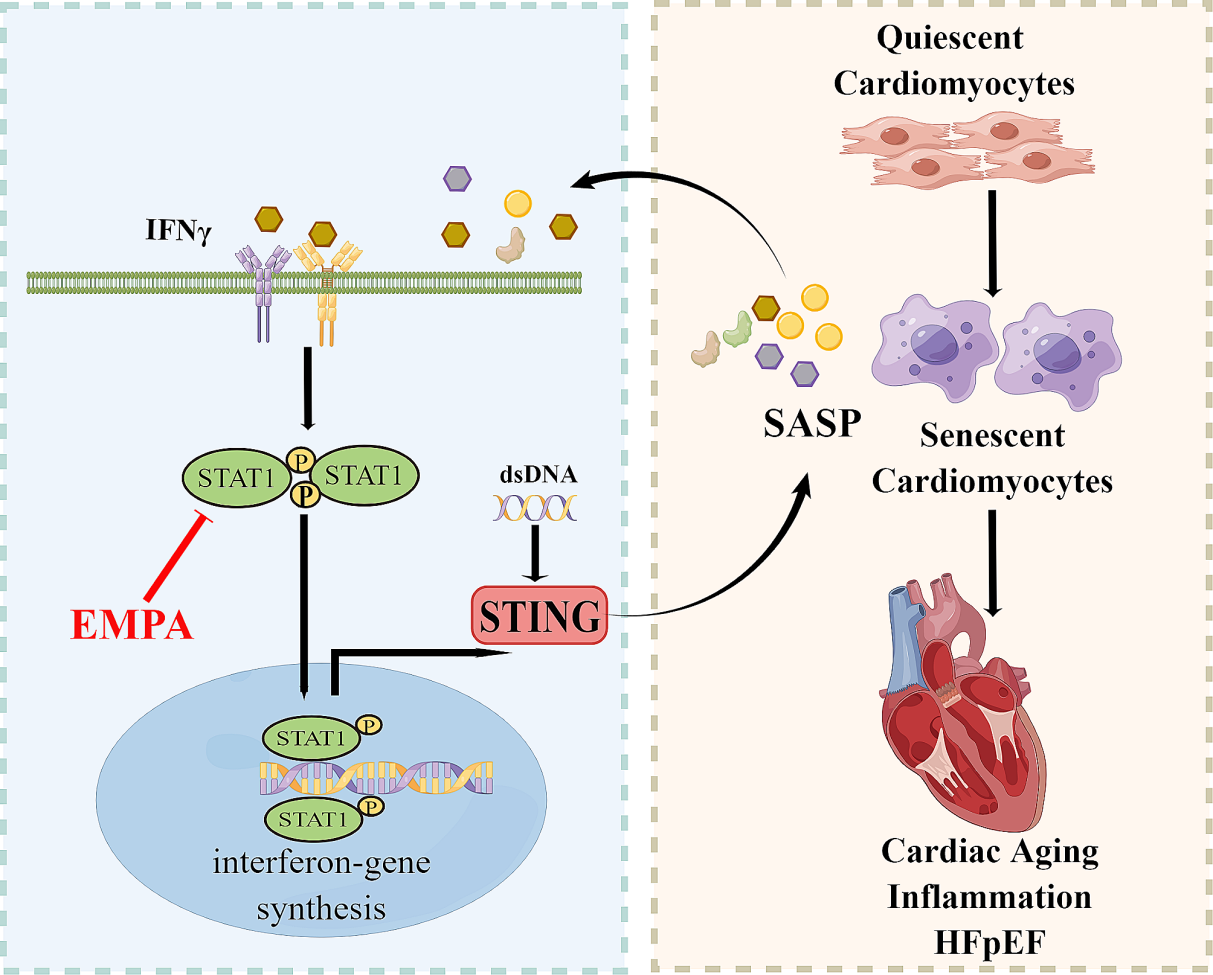
EMPA protects against HFpEF by reducing the inflammation and aging process through the INFγ-STAT1-STING signaling in mice

In summary, our study revealed that EMPA has a beneficial effect on murine HFpEF through reducing oxidative stress and inhibiting inflammatory INFγ-STAT1–STING signaling (Fig. 8). In addition, we found that activation of the STAT1–STING axis promotes cardiomyocyte senescence, which provides a new understanding of how inflammation drives cell senescence. One limitation of this study is the lack of evidence on how EMPA mediates its protective effect via the inhibition of STAT1 in vivo and its causal relationship. In future studies, we will explore the specific role and mechanism of the STAT1–STING axis in HFpEF using gene knockout mice. In addition, female mice should be considered in future studies because sex differences in demographics, comorbidities, and underlying pathophysiology make the phenotypes of HFpEF unique to women [43]. These findings might help in identifying novel therapeutic targets for this prevalent and devastating syndrome. In-depth and rigorous investigations into the interaction between inflammation and aging would be helpful for exploring interventions to delay aging and age-related HFpEF. Importantly, we showed that inhibiting the STAT1–STING axis may be a therapeutic approach for counteracting the aging- and inflammation-associated HFpEF. Our data suggest that EMPA may have potential anti-aging effects, providing additional health benefits for patients with diabetes, heart failure or other age-related diseases.

### Supplementary material

Supplementary Material 1.

Supplementary Material 2.

## Data Availability

The datasets supporting the conclusions of this article are available from the corresponding author upon reasonable request.

## References

[1] Bloom MW, Greenberg B, Jaarsma T, Januzzi JL, Lam CSP, Maggioni AP, Trochu J-N, Butler J. Heart failure with reduced ejection fraction. Nat Rev Dis Prim 2017;3(1):17058. 10.1038/nrdp.2017.58 28836616

[2] Shah SJ, Borlaug BA, Kitzman DW, McCulloch AD, Blaxall BC, Agarwal R, Chirinos JA, Collins S, Deo RC, Gladwin MT, et al. Research priorities for heart failure with preserved ejection fraction. Circulation. 2020;141(12):1001–26. 32202936 10.1161/CIRCULATIONAHA.119.041886 PMC7101072

[3] Withaar C, Li S, Meems LMG, Silljé HHW, de Boer RA. Aging and HFpEF: are we running out of time? J Mol Cell Cardiol. 2022;168:33–4. 35421401 10.1016/j.yjmcc.2022.04.006

[4] Anker SD, Butler J, Filippatos G, Ferreira JP, Bocchi E, Böhm M, Brunner-La Rocca HP, Choi DJ, Chopra V, Chuquiure-Valenzuela E, et al. Empagliflozin in Heart failure with a preserved ejection fraction. N Engl J Med. 2021;385(16):1451–61. 34449189 10.1056/NEJMoa2107038

[5] Solomon SD, McMurray JJV, Claggett B, de Boer RA, DeMets D, Hernandez AF, Inzucchi SE, Kosiborod MN, Lam CSP, Martinez F, et al. Dapagliflozin in heart failure with mildly reduced or preserved ejection fraction. N Engl J Med. 2022;387(12):1089–98. 36027570 10.1056/NEJMoa2206286

[6] Ostrominski JW, Vaduganathan M. Clinical and mechanistic potential of sodium–glucose co-transporter 2 (SGLT2) inhibitors in heart failure with preserved ejection fraction. Am J Med. 2024;137:S9–24. 37160196 10.1016/j.amjmed.2023.04.035

[7] De Lorenzi AB, Kaplinsky E, Zambrano MR, Chaume LT, Rosas JM. Emerging concepts in heart failure management and treatment: focus on SGLT2 inhibitors in heart failure with preserved ejection fraction. Drugs Context 2023, 12:2022-7-1. 10.7573/dic.2022-7-1 PMC9828870 36660013

[8] Loffredo FS, Nikolova AP, Pancoast JR, Lee RT. Heart failure with preserved ejection fraction. Circ Res. 2014;115(1):97–107. 24951760 10.1161/CIRCRESAHA.115.302929 PMC4094348

[9] Childs BG, Gluscevic M, Baker DJ, Laberge R-M, Marquess D, Dananberg J, van Deursen JM. Senescent cells: an emerging target for diseases of ageing. Nat Rev Drug Discov. 2017;16(10):718–35. 28729727 10.1038/nrd.2017.116 PMC5942225

[10] Gevaert AB, Shakeri H, Leloup AJ, Van Hove CE, De Meyer GRY, Vrints CJ, Lemmens K, Van Craenenbroeck EM. Endothelial senescence contributes to heart failure with preserved ejection fraction in an aging mouse model. Circ Heart Fail. 2017;10(6):e003806. 28611124 10.1161/CIRCHEARTFAILURE.116.003806

[11] Li X, Li C, Zhang W, Wang Y, Qian P, Huang H. Inflammation and aging: signaling pathways and intervention therapies. Signal Transduct Target Ther. 2023;8(1):239. 37291105 10.1038/s41392-023-01502-8 PMC10248351

[12] Scisciola L, Olivieri F, Ambrosino C, Barbieri M, Rizzo MR, Paolisso G. On the wake of metformin: do anti-diabetic SGLT2 inhibitors exert anti-aging effects? Ageing Res Rev. 2023;92:102131. 37984626 10.1016/j.arr.2023.102131

[13] La Grotta R, Frigé C, Matacchione G, Olivieri F, de Candia P, Ceriello A, Prattichizzo F. Repurposing SGLT-2 inhibitors to target aging: available evidence and molecular mechanisms. Int J Mol Sci. 2022;23(20):12325. 36293181 10.3390/ijms232012325 PMC9604287

[14] O’Keefe JH, Weidling R, O’Keefe EL, Franco WG. SGLT inhibitors for improving healthspan and lifespan. Prog Cardiovasc Dis. 2023;81:2–9. 37852518 10.1016/j.pcad.2023.10.003 PMC10831928

[15] Fang R, Chen J, Long J, Zhang B, Huang Q, Li S, Li K, Chen Q, Liu D. Empagliflozin improves kidney senescence induced by D-galactose by reducing sirt1-mediated oxidative stress. Biogerontology. 2023;24(5):771–82. 37227544 10.1007/s10522-023-10038-x

[16] Schiattarella GG, Altamirano F, Tong D, French KM, Villalobos E, Kim SY, Luo X, Jiang N, May HI, Wang ZV, et al. Nitrosative stress drives heart failure with preserved ejection fraction. Nature. 2019;568(7752):351–6. 30971818 10.1038/s41586-019-1100-z PMC6635957

[17] Shi Y, Zhao L, Wang J, Liu S, Zhang Y, Qin Q. The selective NLRP3 inflammasome inhibitor MCC950 improves isoproterenol-induced cardiac dysfunction by inhibiting cardiomyocyte senescence. Eur J Pharmacol. 2022;937:175364. 36336012 10.1016/j.ejphar.2022.175364

[18] Zannad F, Ferreira JP, Butler J, Filippatos G, Januzzi JL, Sumin M, Zwick M, Saadati M, Pocock SJ, Sattar N, et al. Effect of empagliflozin on circulating proteomics in heart failure: mechanistic insights into the EMPEROR programme. Eur Heart J. 2022;43(48):4991–5002. 36017745 10.1093/eurheartj/ehac495 PMC9769969

[19] Hahn VS, Petucci C, Kim M-S, Bedi KC, Wang H, Mishra S, Koleini N, Yoo EJ, Margulies KB, Arany Z, et al. Myocardial metabolomics of Human Heart failure with preserved ejection fraction. Circulation. 2023;147(15):1147–61. 36856044 10.1161/CIRCULATIONAHA.122.061846 PMC11059242

[20] Mustroph J, Lücht CM, Wagemann O, Sowa T, Hammer KP, Sag CM, Tarnowski D, Holzamer A, Pabel S, Beuthner BE, et al. Empagliflozin enhances human and murine cardiomyocyte glucose uptake by increased expression of GLUT1. Diabetologia. 2019;62(4):726–9. 30694352 10.1007/s00125-019-4819-z

[21] Valero-Muñoz M, Saw EL, Hekman RM, Blum BC, Hourani Z, Granzier H, Emili A, Sam F. Proteomic and phosphoproteomic profiling in heart failure with preserved ejection fraction (HFpEF). Front Cardiovasc Med. 2022;9:966968. 36093146 10.3389/fcvm.2022.966968 PMC9452734

[22] Pena E, El Alam S, Siques P, Brito J. Oxidative stress and diseases associated with high-altitude exposure. Antioxidants (Basel). 2022;11(2):267. 35204150 10.3390/antiox11020267 PMC8868315

[23] Ashour D, Rebs S, Arampatzi P, Saliba A-E, Dudek J, Schulz R, Hofmann U, Frantz S, Cochain C, Streckfuß-Bömeke K, et al. An interferon gamma response signature links myocardial aging and immunosenescence. Cardiovasc Res. 2023;119(14):2458–68. 37141306 10.1093/cvr/cvad068 PMC10651211

[24] Mills RJ, Humphrey SJ, Fortuna PRJ, Lor M, Foster SR, Quaife-Ryan GA, Johnston RL, Dumenil T, Bishop C, Rudraraju R, et al. BET inhibition blocks inflammation-induced cardiac dysfunction and SARS-CoV-2 infection. Cell. 2021;184(8):2167–e21822122. 33811809 10.1016/j.cell.2021.03.026 PMC7962543

[25] Bhattacharyya R, Gupta P, Bandyopadhyay SK, Patro BS, Chattopadhyay S. Coralyne, a protoberberine alkaloid, causes robust photosenstization of cancer cells through ATR-p38 MAPK-BAX and JAK2-STAT1-BAX pathways. Chem Biol Interact. 2018;285:27–39. 29486184 10.1016/j.cbi.2018.02.032

[26] Victorelli S, Salmonowicz H, Chapman J, Martini H, Vizioli MG, Riley JS, Cloix C, Hall-Younger E, Machado Espindola-Netto J, Jurk D, et al. Apoptotic stress causes mtDNA release during senescence and drives the SASP. Nature. 2023;622(7983):627–36. 37821702 10.1038/s41586-023-06621-4 PMC10584674

[27] Gulen MF, Samson N, Keller A, Schwabenland M, Liu C, Glück S, Thacker VV, Favre L, Mangeat B, Kroese LJ, et al. cGAS–STING drives ageing-related inflammation and neurodegeneration. Nature. 2023;620(7973):374–80. 37532932 10.1038/s41586-023-06373-1 PMC10412454

[28] Kandhaya-Pillai R, Yang X, Tchkonia T, Martin GM, Kirkland JL, Oshima J. TNF-α/IFN-γ synergy amplifies senescence-associated inflammation and SARS-CoV-2 receptor expression via hyper-activated JAK/STAT1. Aging Cell. 2022;21(6):e13646. 35645319 10.1111/acel.13646 PMC9197409

[29] Giannattasio S, Citarella A, Trocchianesi S, Filardi T, Morano S, Lenzi A, Ferretti E, Crescioli C. Cell-target-specific anti-inflammatory effect of Empagliflozin: in Vitro evidence in human cardiomyocytes. Front Mol Biosci. 2022;9:879522. 35712355 10.3389/fmolb.2022.879522 PMC9194473

[30] Omrani O, Krepelova A, Rasa SMM, Sirvinskas D, Lu J, Annunziata F, Garside G, Bajwa S, Reinhardt S, Adam L, et al. IFNγ-Stat1 axis drives aging-associated loss of intestinal tissue homeostasis and regeneration. Nat Commun. 2023;14(1):6109. 37777550 10.1038/s41467-023-41683-y PMC10542816

[31] Decout A, Katz JD, Venkatraman S, Ablasser A. The cGAS–STING pathway as a therapeutic target in inflammatory diseases. Nat Rev Immunol. 2021;21(9):548–69. 33833439 10.1038/s41577-021-00524-z PMC8029610

[32] Luo W, Zou X, Wang Y, Dong Z, Weng X, Pei Z, Song S, Zhao Y, Wei Z, Gao R, et al. Critical role of the cGAS–STING pathway in doxorubicin-induced cardiotoxicity. Circ Res. 2023;132(11):e223-42. 37154056 10.1161/CIRCRESAHA.122.321587

[33] Wang X, Yang C, Wang X, Miao J, Chen W, Zhou Y, Xu Y, An Y, Cheng A, Ye W, et al. Driving axon regeneration by orchestrating neuronal and non-neuronal innate immune responses via the IFNγ-cGAS–STING axis. Neuron. 2023;111(2):236–e255237. 36370710 10.1016/j.neuron.2022.10.028 PMC9851977

[34] Madani AY, Majeed Y, Abdesselem HB, Agha MV, Vakayil M, Sukhun NKA, Halabi NM, Kumar P, Hayat S, Elrayess MA, et al. Signal transducer and activator of transcription 3 (STAT3) suppresses STAT1/interferon signaling pathway and inflammation in senescent preadipocytes. Antioxidants (Basel). 2021;10(2):334. 33672392 10.3390/antiox10020334 PMC7927067

[35] Ma F, Li B, Yu Y, Iyer SS, Sun M, Cheng G. Positive feedback regulation of type I interferon by the interferon-stimulated gene STING. EMBO Rep. 2015;16(2):202–12. 25572843 10.15252/embr.201439366 PMC4328747

[36] Dong G, You M, Ding L, Fan H, Liu F, Ren D, Hou Y. STING negatively regulates double-stranded DNA-activated JAK1-STAT1 signaling via SHP-1/2 in B cells. Mol Cells. 2015;38(5):441–51. 25947293 10.14348/molcells.2015.2359 PMC4443286

[37] Preda A, Montecucco F, Carbone F, Camici GG, Lüscher TF, Kraler S, Liberale L. SGLT2 inhibitors: from glucose-lowering to cardiovascular benefits. Cardiovasc Res. 2024;120(5):443–60. 38456601 10.1093/cvr/cvae047 PMC12001887

[38] Scisciola L, Taktaz F, Fontanella RA, Pesapane A, Surina, Cataldo V, Ghosh P, Franzese M, Puocci A, Paolisso P, et al. Targeting high glucose-induced epigenetic modifications at cardiac level: the role of SGLT2 and SGLT2 inhibitors. Cardiovasc Diabetol. 2023;22(1):24. 36732760 10.1186/s12933-023-01754-2 PMC9896756

[39] Scisciola L, Chianese U, Caponigro V, Basilicata MG, Salviati E, Altucci L, Campiglia P, Paolisso G, Barbieri M, Benedetti R, et al. Multi-omics analysis reveals attenuation of cellular stress by empagliflozin in high glucose-treated human cardiomyocytes. J Transl Med. 2023;21(1):662. 37742032 10.1186/s12967-023-04537-1 PMC10518098

[40] Gao Y-M, Feng S-T, Wen Y, Tang T-T, Wang B, Liu B-C. Cardiorenal protection of SGLT2 inhibitors-perspectives from metabolic reprogramming. EBioMedicine. 2022;83:104215. 35973390 10.1016/j.ebiom.2022.104215 PMC9396537

[41] Rasa SMM, Annunziata F, Krepelova A, Nunna S, Omrani O, Gebert N, Adam L, Käppel S, Höhn S, Donati G et al. Inflammaging is driven by upregulation of innate immune receptors and systemic interferon signaling and is ameliorated by dietary restriction. Cell Rep 2022;39(13):111017. 10.1016/j.celrep.2022.111017 35767948

[42] Giannattasio S, Citarella A, Trocchianesi S, Filardi T, Morano S, Lenzi A, Ferretti E, Crescioli C. Cell-target-specific anti-inflammatory effect of Empagliflozin: in Vitro evidence in human cardiomyocytes. Front Mol Biosci. 2022;9:879522. 35712355 10.3389/fmolb.2022.879522 PMC9194473

[43] Tibrewala A, Yancy CW. Heart failure with preserved ejection fraction in women. Heart Fail Clin. 2019;15(1):9–18. 30449384 10.1016/j.hfc.2018.08.002

