# Supplementary material for: Empagliflozin protects against heart failure with preserved ejection fraction partly by inhibiting the senescence-associated STAT1–STING axis
Source: Cardiovasc Diabetol. 2024 Jul 23;23:269. doi: 10.1186/s12933-024-02366-0 (PMC11267814; doi:10.1186/s12933-024-02366-0)
Supplement: Supplementary file 2 — Supplementary Material 2. [file 12933_2024_2366_MOESM2_ESM.pdf]

Supplemental Fig. 1

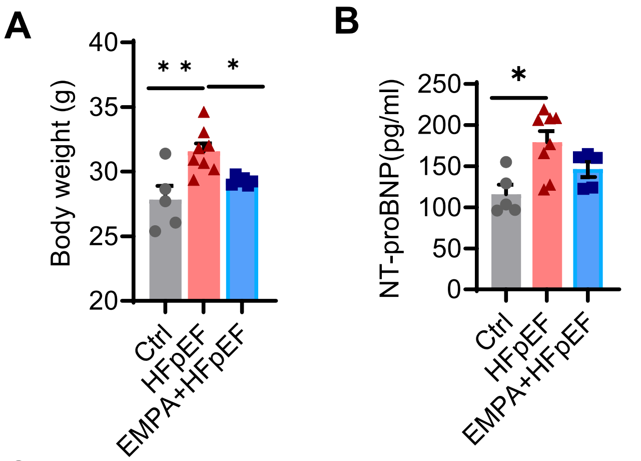

C

	Control	HFpEF	EMPA+HFpEF
Heart rate (bpm)	493.80±30.73	526±37.71	530.00±21.21
Ejection Fraction (%)	49.62±3.17	52.56±2.56	53.99±5.18
E/E' ration	20.72±1.03	31.86±3.20**	23.84±1.96#
LVID;d (mm)	3.53±0.19	3.47±0.02	3.54±0.31
LVPW;d (mm)	0.68±0.02	0.83±0.04*	0.71±0.04#
LVID;s (mm)	2.87±0.38	2.91±0.29	2.63±0.14

Supplemental Fig. 1 (A) Body weight (BW) in different groups, n=7; (B) The serum levels of NT-proBNP in different groups, n=5-8. (C) Echocardiographic parameters in different groups, n=5-8, ***P*<0.01 vs control, . #*P*<0.05 vs HFpEF; LVID: Left ventricular internal diameter; LVPW: left ventricular posterior wall (d = diastolic; s = systolic). The data are shown as means ± SEM, One-way ANOVA with Tukey's multiple comparisons test was used for comparisons, **P*<0.05, ***P*<0.01 .

.

Supplemental Fig. 2

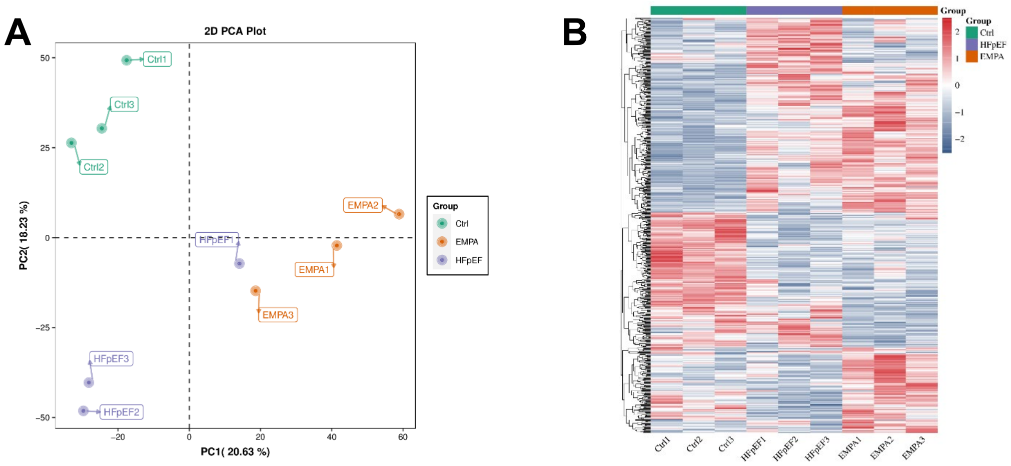

Supplemental Fig. 2 4D-DIA quantitative proteomics analysis was performed at the heart tissue level in Control, HFpEF and EMPA-treated mice. (A) Principal component (PC) analysis based on all myocardial proteins from the three groups; (B) Heatmap of the differentially expressed proteins (DEPs).

Supplemental Fig. 3

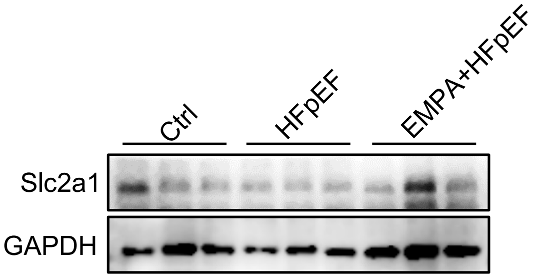

Supplemental Fig. 3 Representative western blot image of Slac2a1 (GLUT1) in the hearts.

Supplemental Fig. 4

A

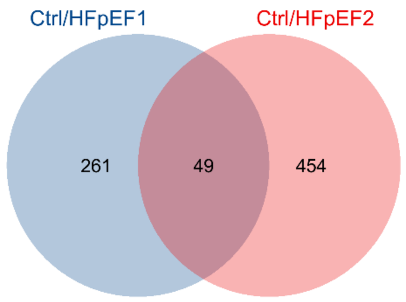

B

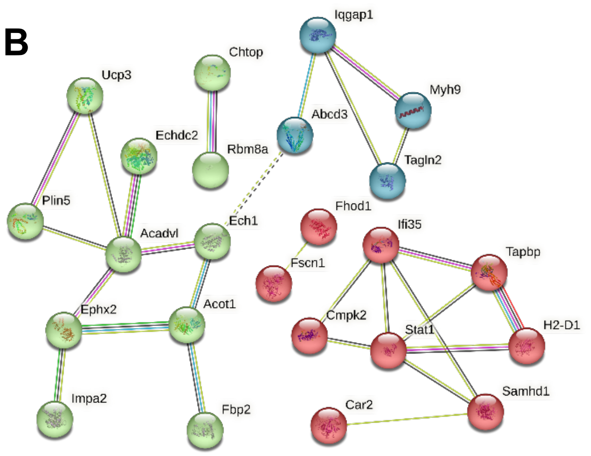

C

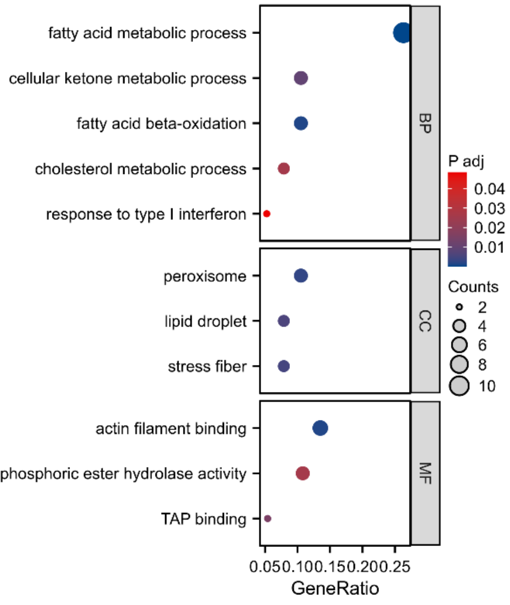

D

Gene	DESCRIPTION	FC1	Pvalue1	FC2	Pvalue2
Cnpy2	Protein canopy homolog 2	1.25	0.0166	1.68	0.0002
Myh9	Myosin-9	1.22	0.008	1.27	0.0004
Fhod1	Fascin	1.28	0.0359	1.57	0.0009
Vwa8	von Willebrand factor A domain-containing protein 8	1.56	0.0211	1.20	0.0016
Me1	NADP-dependent malic enzyme	1.36	0.0047	1.17	0.0021
**Stat1**	Signal transducer and activator of transcription 1	1.56	0.0144	1.58	0.0028
Ephx2	Bifunctional epoxide hydrolase 2;Cytosolic epoxide hydrolase 2;Lipid-phosphate phosphatase	1.60	0.003	1.24	0.0038
Snf8	Vacuolar-sorting protein SNF8	1.26	0.0425	1.21	0.0062
Col18a1	Collagen alpha-1(XVIII) chain;Endostatin	1.65	0.0103	1.14	0.0068
Acot1	Acyl-coenzyme A thioesterase 1	2.75	0.0091	1.32	0.0069

Supplemental Fig. 4 Bioinformatics analysis of proteomic results from the hearts in different HFpEF models induced by HFD+L-NAME regimen and salty drinking water and unilateral nephrectomy combined with chronic exposure to aldosterone. (A) The venn diagram of DEPs beween Ctrl/HFpEF1 and Ctrl/HFpEF2; (B) The interaction diagram of coregulated proteins; (C) GO enrichment of coregulated proteins; (D) Representive proteins among the coregulated proteins. HFpEF1: HFpEF model induced by HFD+L-NAME in our study; HFpEF2: HFpEF model induced by salty drinking water and unilateral nephrectomy combined with chronic exposure to aldosterone.

Supplemental Fig. 5

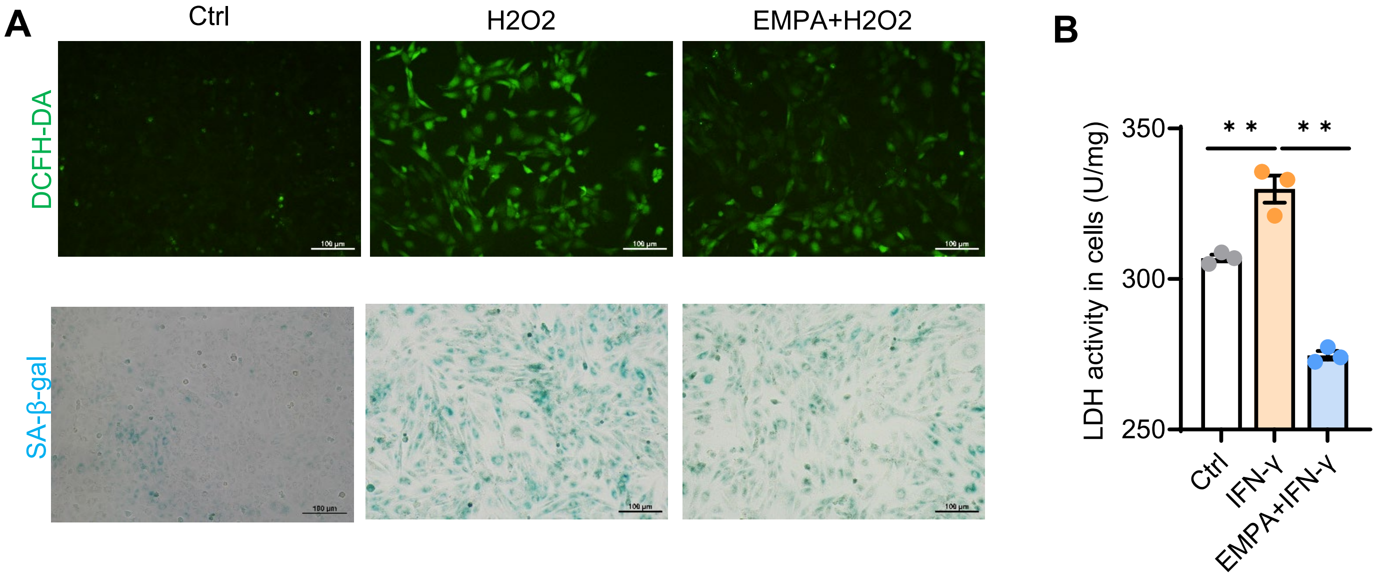

Supplemental Fig. 5 (A) Representative images of DCFH-DA staining (top) and SA-β-gal staining (bottom); (B) The LDH activity n H9C2 cells treated with IFN-γ and EMPA pretreatment, n=3. The data are shown as means ± SEM, One-way ANOVA with Tukey's multiple comparisons test was used for comparisons, *P<0.05, **P<0.01

Supplemental Fig. 6

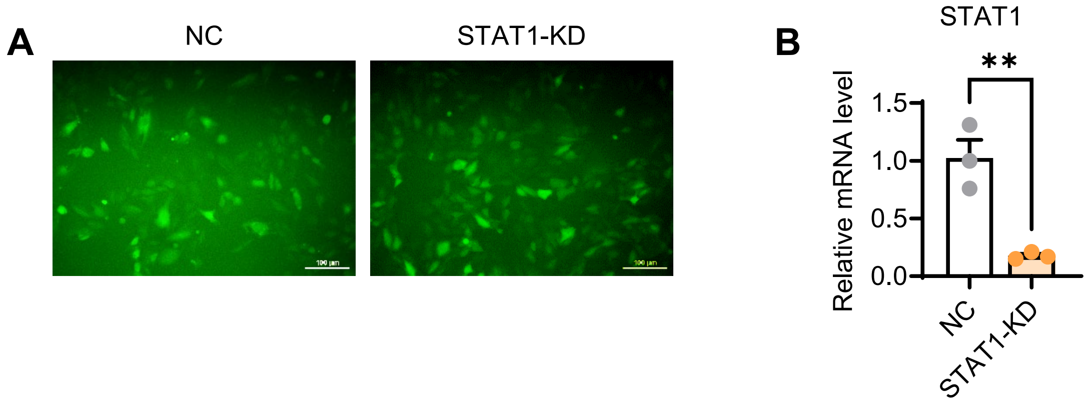

Supplemental Fig. 6 The stable cell line with STAT1-knockdown were established using the recombinant lentivirus system (A) Representative fluorescent images in H9C2 cells after transfection, the green colour indicated GFP; (B) The efficiency of knockdown was verified by RT-PCR, n=3. The data are shown as means ± SEM, unpaired Student's t test was used for comparisons, **P<0.01.

Supplemental Fig. 7

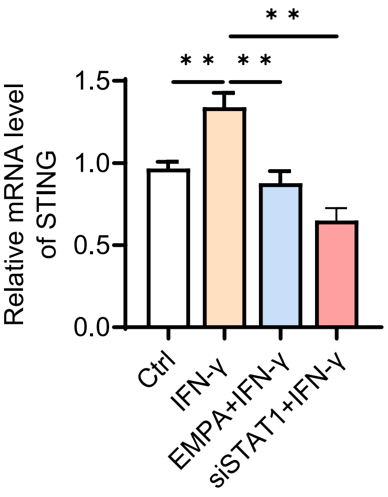

Supplemental Fig. 7 mRNA levels of STING in H9C2 cells treated with EMPA or siSTAT1 after IFNγ administration, n=3. All data are shown as means ± SEM; One-way ANOVA with Tukey's multiple comparisons test or unpaired Student's t-test was used for comparisons, *P<0.05, **P<0.01.
